# Supplementary material for: Platelet Toll-like Receptor 4–Related Innate Immunity Potentially Participates in Transfusion Reactions Independent of ABO Compatibility: An Ex Vivo Study
Source: Biomedicines. 2021 Dec 23;10(1):29. doi: 10.3390/biomedicines10010029 (PMC8772939; doi:10.3390/biomedicines10010029)
Supplement: Supplementary file 1 [file biomedicines-10-00029-s001.zip › biomedicines-1489661-supplementary.pdf]

Supplementary file

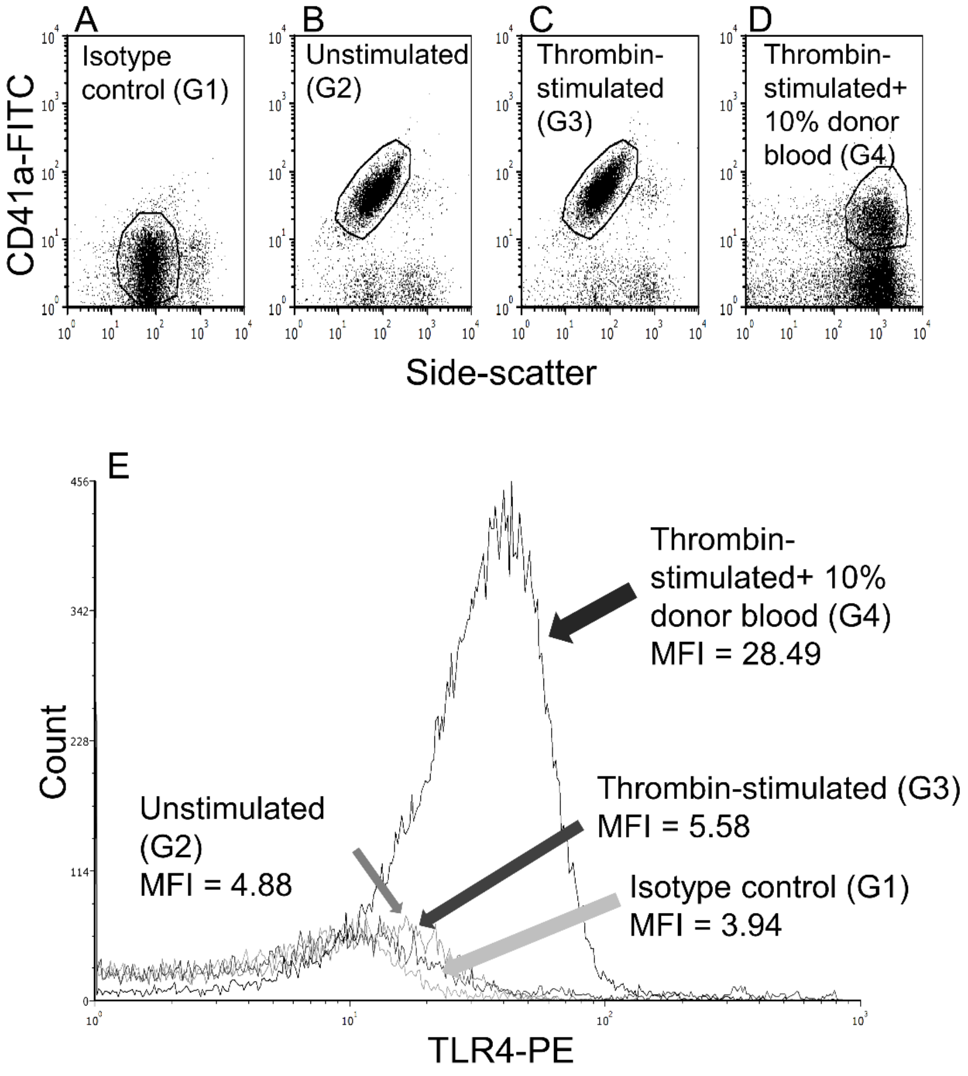

Figure S1. Flow cytometry for platelet TLR4 expression. Individual platelet events are revealed by (on the x-axis) their side-scatter properties (granularity) and (on the y-axis) their positive labeling with platelet-specific monoclonal antibodies (CD41a- FITC). Dot plots show the fluorescence of the isotype control (A), unstimulated platelets (B), and platelets stimulated with thrombin without (C) and with 10% donor blood (v/v) (D). Histogram exhibiting the platelets' fluorescence (TLR4-PE; x-axis) (E). MFI, mean fluorescence intensity.

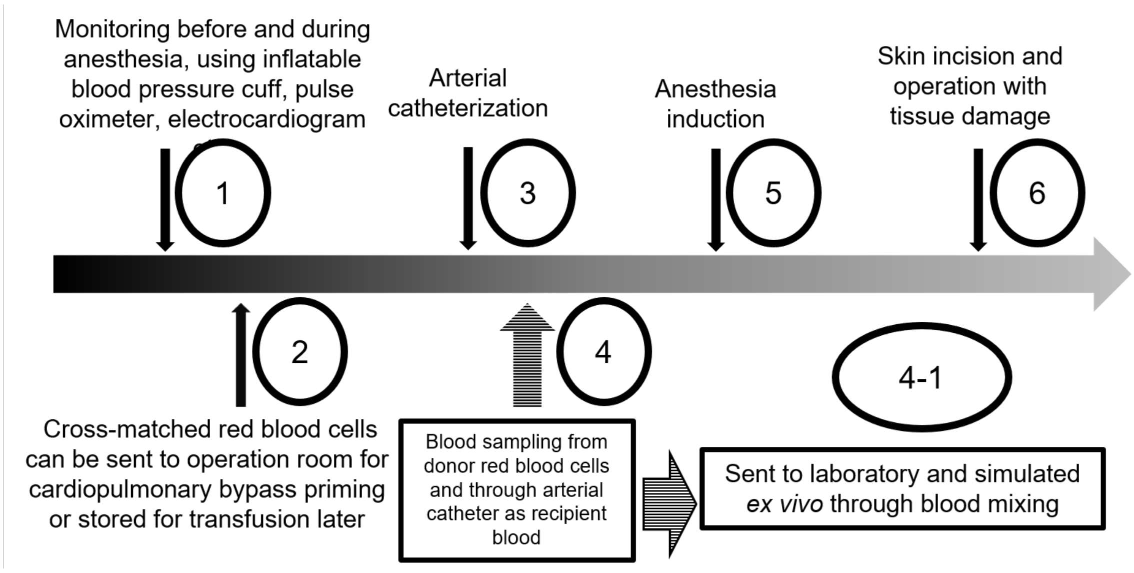

Figure S2. Key steps of general anesthesia and cardiac surgery performed. First, essential hemodynamic monitoring was ensured. Second, cross-matched red blood cells were sent to the operation room. Subsequently, arterial catheterization was performed to obtain recipient blood samples. Anesthetics were administered, and skin incisions were applied. We reveal that theoretically, blood sampling before anesthesia induction and skin incision eliminates influence from confounding factors related to anesthetics and tissue damage–related factors in the blood circulation system.
